# An Insight into *Sargassum muticum* Cytoprotective Mechanisms against Oxidative Stress on a Human Cell In Vitro Model

**DOI:** 10.3390/md15110353

**Published:** 2017-11-10

**Authors:** Susete Pinteus, Marco F. L. Lemos, Joana Silva, Celso Alves, Agnieszka Neugebauer, Rafaela Freitas, Adriana Duarte, Rui Pedrosa

**Affiliations:** MARE—Marine and Environmental Sciences Centre, School of Tourism and Maritime Technology, Polytechnic Institute of Leiria, 2520-641 Peniche, Portugal; susete.pinteus@ipleiria.pt (S.P.); marco.lemos@ipleiria.pt (M.F.L.L.); joana.m.silva@ipleiria.pt (J.S.); celso.alves@ipleiria.pt (C.A.); azneugebauer@gmail.com (A.N.); rafaela.freitas@ipleiria.pt (R.F.); adrianaduarte.1996@hotmail.com (A.D.)

**Keywords:** antioxidant compounds, brown algae, Caspase-9, cell death mechanisms, cytoprotection, marine natural compounds, MCF-7 cells, mitochondrial membrane potential, reactive oxygen species (ROS), seaweed

## Abstract

*Sargassum muticum* is a brown seaweed with strong potential to be used as a functional food ingredient, mainly due to its antioxidant properties. It is widely used in traditional oriental medicine for the treatment of numerous diseases. Nevertheless, few studies have been conducted to add scientific evidence on its effects as well as on the mechanisms of action involved. In this work, the human cell line MCF-7 was used as an in vitro cellular model to evaluate the capability of *Sargassum muticum* enriched fractions to protect cells on an oxidative stress condition. The concentration of the bioactive compounds was obtained by vacuum liquid chromatography applied on methanol (M) and 1:1 methanol:dichloromethane (MD) crude extracts, resulting in seven enriched fractions from the M extraction (MF2–MF8), and eight fractions from the MD extraction (MDF1–MDF8). All fractions were tested for cytotoxic properties on MCF-7 cells and the nontoxic ones were tested for their capacity to blunt the damaging effects of hydrogen peroxide-induced oxidative stress. The nontoxic effects were also confirmed in 3T3 fibroblast cells as a nontumor cell line. The antioxidant potential of each fraction, as well as changes in the cell’s real-time hydrogen peroxide production, in the mitochondrial membrane potential, and in Caspase-9 activity were evaluated. The results suggest that the protective effects evidenced by *S. muticum* can be related with the inhibition of hydrogen peroxide production and the inhibition of Caspase-9 activity.

## 1. Introduction

There has been a paradigm change in consumer behavior associated with the increasing knowledge that health is directly related with food quality. In fact, it is now evident that healthy food, rich in vitamins and minerals, can prevent or delay the development of several oxidative stress associated diseases [1,2]. Biological systems produce different oxidative molecules that in normal conditions are balanced by enzymatic and nonenzymatic defenses. However, due to several factors, such as stress, smoking and drinking, pollution, and poor food intake quality, the production of oxidative molecules increases promoting an imbalance between these molecules and the antioxidant defense mechanisms. Reactive oxygen species (ROS) include unstable metabolites of molecular oxygen (O_2_) that have higher reactivity than O_2_—such as the superoxide radical (O_2_^•−^) and the hydroxyl radical (HO^•^), and nonradical molecules such as hydrogen peroxide (H_2_O_2_). These highly reactive molecules often oxidize important biological molecules such as lipids, DNA, and proteins, producing toxic substances that accumulate in cells leading to pathologies such as diabetes, neurodegenerative diseases, cardiovascular diseases, and cancer, among others [1]. Since many of these diseases do not have an effective cure, it is of upmost importance to search for new compounds that may be used to decrease oxidative stress conditions.

For thousands of years, seaweed have been valued and widely consumed as a direct human food in many Asian countries [3]. Indeed, many studies address the lower incidence of diseases like cancer and diabetes in these countries, which can be directly related to dietary habits [4,5]. Due to this evidence, and an increasing need for new natural products for drug development, in the last decades, seaweeds have been targeted for bioactive compounds discovery, and many important bioactivities have been recognized, such as antitumor, anti-inflammatory, antioxidant, and antimicrobial activities [6,7,8,9,10]. Within seaweed, brown seaweeds (Heterokontophyta) are recognized for being the exclusive producers of potent antioxidant molecules—the phlorotannins. However, they also contain other important compounds such as fatty acids, terpenoids, carotenoids, sterols, and polysaccharides [11,12,13,14]. *Sargassum muticum* is a brown seaweed belonging to the Fucales, being native from Asia. Since the 1940s and 1970s, *S. muticum* has been recorded in North America and Europe, respectively, where it is currently widely used, presenting a highly invasive behavior [15,16,17,18]. Despite *Sargassum muticum* being a widely known species, few studies have been conducted addressing its biological properties. Nevertheless, within the Sargassaceae family, some interesting studies can be found reporting different biological activities [10,19] such as the potential to inhibit acetylcholinesterase activity [20] and also neuroprotective effects. In addition, pheophytin A, purified from the Japanese brown alga *Sargassum fulvellum*, was reported as a novel neurodifferentiation compound [21], and sargachromenol isolated from *Sargassum macrocarpum* was shown to markedly promote novel nerve growth in PC12D cells [22]. Zandi and co-workers [23] also reported that the cold water extract from *Sargassum oligocystum* presents anticancer activity against tumor cells replication.

While some studies point out phlorotannins as the main bioactive compounds in brown seaweeds, other studies have proved that compounds such as sulfated polyssacharides, sterols, sargaquinoic acids, and carotenoids, have also shown diverse biological activities like analgesic, anti-inflammatory, antioxidant, neuroprotective, antimicrobial, antitumor, fibrinolytic, immune-modulatory, anticoagulant, hepatoprotective, and antiviral activity, among others [10]. For instance, polysaccharides obtained from *Sargassum fusiforme* showed significant antitumor activity both in vitro and in vivo, and improved the immune function in tumor-bearing mice [24]. By other side, sulfated polysaccharides from *Sargassum fulvellum* showed anticoagulant activity [25].

Although the uses of *Sargassum* species in Occidental countries have been scarcely valued (used mainly as fertilizer, for animal feed, and for alginate extraction) [3], in Asia the empirical use of *Sargassum* species for the treatment of a variety of diseases including skin and thyroid diseases, arteriosclerosis, bronchitis, among others, is a common practice [19]. Despite these uses, there is a lack of scientific evidence of its pharmacological properties, with scarce data existing on its effective results as well as the mechanisms of action. *Sargassum* *muticum* is known to produce molecules with potent antioxidant properties [8,26,27], however, it is important to understand if it has potential to protect human cells on oxidative stress conditions.

The aim of this study was to evaluate if the fractions of *Sargassum muticum*, obtained by vacuum liquid chromatography (VLC), presented antioxidant and cytoprotective potential on human cells (MCF-7 cells) when they are exposed to H_2_O_2_, as an oxidative stress condition, and also to unravel the potential mechanisms associated to the observed effects by addressing mitochondrial membrane potential and Caspase-9 activity. Through this approach, this research can add evidence of this seaweed’s ethnopharmaceutical properties, pointing out to the nutraceutical potential of this abundant marine resource.

## 2. Results

### 2.1. Antioxidant Activity

To understand the antioxidant potential of each fraction, the total phenolic content (TPC), the oxygen radical absorbance capacity (ORAC), and the 1,1-diphenyl-2-picryl-hydrazyl (DPPH) radical scavenging capacity were evaluated. The results are shown in Table 1.

In both VLCs, the last fraction, F8, presented the highest phenolic content, namely 84.080 ± 0.026 and 61.668 ± 1.496 mg PE/g extract, for methanol:dichloromethane (MD) F8 and methanol (M) F8, respectively. Regarding the DPPH radical scavenging ability, within the MD-VLC, fractions MDF5–MDF8 presented the highest potential, and within the M-VLC, fractions MF2, MF3, and MF8 presented the highest potential. In both chromatographies, fraction F8 was the most potent with an IC_50_ of 32.29 (28.95–36.02) and 36.4 (32.4–41.0) µg/mL for MDF8 and MF8, respectively. Concerning oxygen radical absorbance capacity, the results suggest that, within the MD-VLC, the antioxidant activity increases along fractions, where the MDF8 fraction was found to be the most potent with 3040.143 ± 266.235 µmol TE/g extract. Concerning the M-VLC fractions, the results revealed a weak oxygen radical absorbance capacity transversely to all fractions, with the strongest potential exhibited by MF5, with 77.877 ± 22.648 µmol TE/g extract.

### 2.2. Protective Effect of Sargassum muticum Fractions on MCF-7 Cells Exposed to H_2_O_2_

To assess the cytoprotective potential of each fraction, their toxicity was evaluated on MCF-7 cells through the 3-(4,5-dimethylthiazol-2-yl)-2,5-diphenyl tetrazolium bromide (MTT) method. The fractions with no cytotoxicity were evaluated for their cytoprotective potential, namely fractions F4, F6, F7, and F8 from the M-VLC, and F1, F2, F3, F4, and F7 obtained from the MD-VLC (see Supplementary Data).

Hydrogen peroxide (H_2_O_2_) is a cytotoxic agent capable of disrupting normal cells functioning leading to cells death. In this assays, cells are coexposed to H_2_O_2_ (0.2 mM) in the presence and absence of the seaweed fractions during 24 h. After this period, cells were washed and their viability verified by evaluating the mitochondrial dehydrogenase activity through the MTT method. 

**Figure 1:**
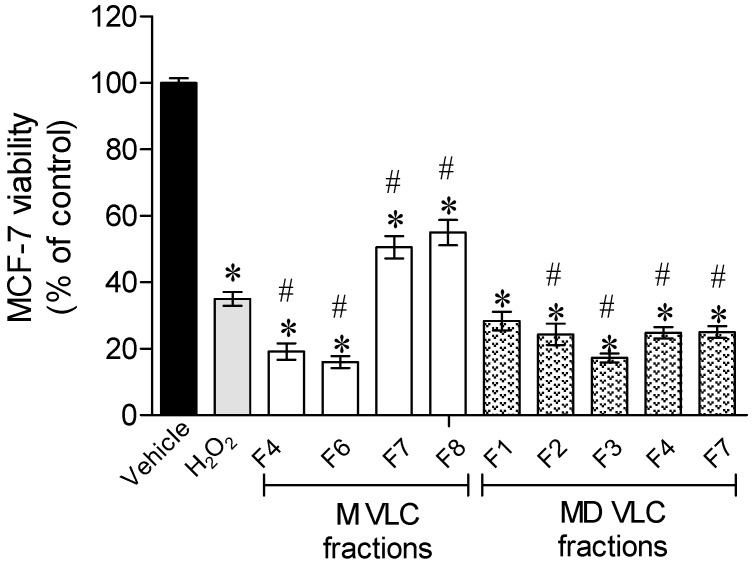
Effect of *Sargassum muticum* fractions (1 mg/mL) in an oxidative stress condition promoted by H_2_O_2_ (0.2 mM), after 24 h of treatment, on MCF-7 cells. Results were obtained by the 3-(4,5-dimethylthiazol-2-yl)-2,5-diphenyl tetrazolium bromide (MTT) method. Values in each column represent the mean ± standard error of the mean of three independent experiments. Symbols represent statistically significant differences (*p* < 0.05, Kruskal Wallis, ANOVA, Dunett’s test) when compared to: * vehicle and ^#^ H_2_O_2_.

The results presented in Figure 1 reveal that when exposing cells to H_2_O_2_, cells viability decreased more than 50%. In the presence of *Sargassum muticum* VLC fractions, two fractions were able to partially revert the H_2_O_2_ effects, namely fractions F7 and F8, both obtained with a methanolic extraction. To understand the relevance of the observed results, the cytotoxicity of MF7 and MF8 fractions were also assessed on the nontumor cells, the primary mouse embryonic fibroblast cells 3T3, revealing no toxicity (MF7—118.7 ± 4.8% of 3T3 viable cells; MF8—121.7 ± 5.7% of 3T3 viable cells).

### 2.3. Cellular Mechanisms Involved in the Cytotoxicity Induced by H_2_O_2_ on MCF-7 Cells in the Presence or Absence of Sargassum muticum Fractions

#### 2.3.1. Real-Time Quantification of H_2_O_2_ Production

Cells produce H_2_O_2_ as a result of metabolic processes. This production may increase under stress conditions. In this assay, the basal H_2_O_2_ production as well as the H_2_O_2_ produced after exposure to this cytotoxic agent, in the presence or absence of *S. muticum* fractions, were evaluated. Therefore, MCF-7 cells were incubated with H_2_O_2_ (0.2 mM) in the presence and absence of *S. muticum* fractions (24 h). The medium was then removed and the Amplex Red together with horseradish peroxidase was added, which in the presence of H_2_O_2_ is converted to resoforin—a highly fluorescent compound. This conversion was accompanied along 60 min allowing the real time quantification of the production of H_2_O_2_.

**Figure 2:**
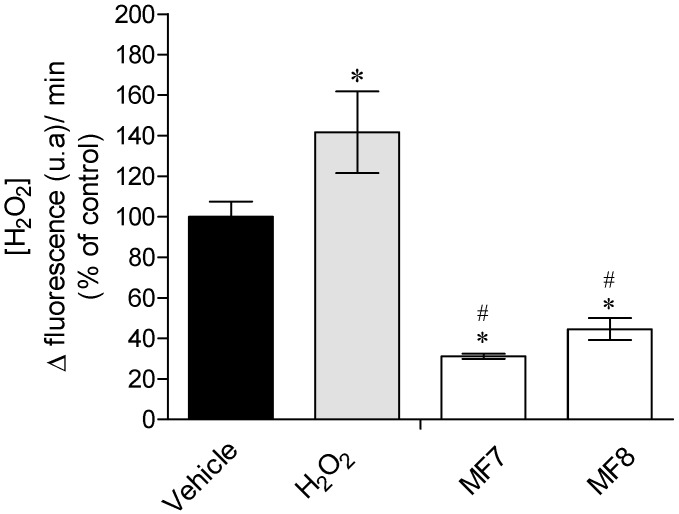
Real time production of H_2_O_2_ by MCF-7 cells in the absence or presence of the MF7 and MF8 (1 mg/mL; 24 h) fractions of *Sargassum muticum*. Each column represent the mean ± standard error of the mean (SEM) of three independent experiments. Symbols represent statistically significant differences (*p* < 0.05, ANOVA, Dunett’s test) when compared to: * vehicle and ^#^ H_2_O_2_.

Results reveal that fractions MF7 and MF8 induced significant differences in the real time H_2_O_2_ production (Figure 2), decreasing the H_2_O_2_ production in about 70 and 56%, respectively, regarding the vehicle situation (basal H_2_O_2_ production), and about 78 and 68.5%, respectively, comparing to the H_2_O_2_ situation. 

#### 2.3.2. Mitochondrial Membrane Potential (ΔΨm)

Mitochondria are the engines of cellular respiration being responsible for adenosine triphosphate (ATP) production, which is mainly regulated by the mitochondrial membrane potential (ΔΨm) generated by an electrochemical gradient across their inner membrane. Therefore, changes in the ΔΨm have been originally proposed as early and obligate events in the apoptotic signaling pathway [28,29].

**Figure 3:**
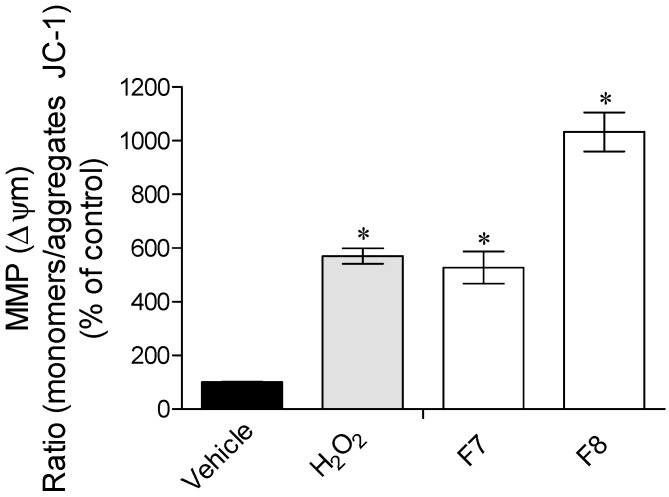
Effects of H_2_O_2_ (0.2 mM) in the presence or absence of *Sargassum muticum* fractions (1 mg/mL) in mitochondrial membrane potential of MCF-7 cells after 24 h of incubation. Results were obtained by the ratio between the monomers/aggregates of JC-1. The values in each column represent the mean ± standard error of the mean (SEM) of three experiments. Symbols represent statistically significant differences (*p* < 0.05, ANOVA, Dunett’s test) when compared to: * vehicle.

The H_2_O_2_ treatment promoted a mitochondrial membrane depolarization (Figure 3). The presence of the seaweed fractions did not reduce the depolarization induced by H_2_O_2_. Additionally, fraction MF8 seemed to potentiate the H_2_O_2_ effects.

#### 2.3.3. Caspase-9 Activity

Caspase-9 is a member of the caspase family of cysteine proteases, a key element in apoptotic events. Once activated, Caspase-9 triggers a succession of reactions known as caspase cascade, leading to cell death by apoptosis. In response to the oxidative stress insult, Caspase-9 is activated, therefore it is appropriate to understand if the cellular protection evidenced by *S. muticum* fractions is due to the inhibition of this enzyme. 

**Figure 4:**
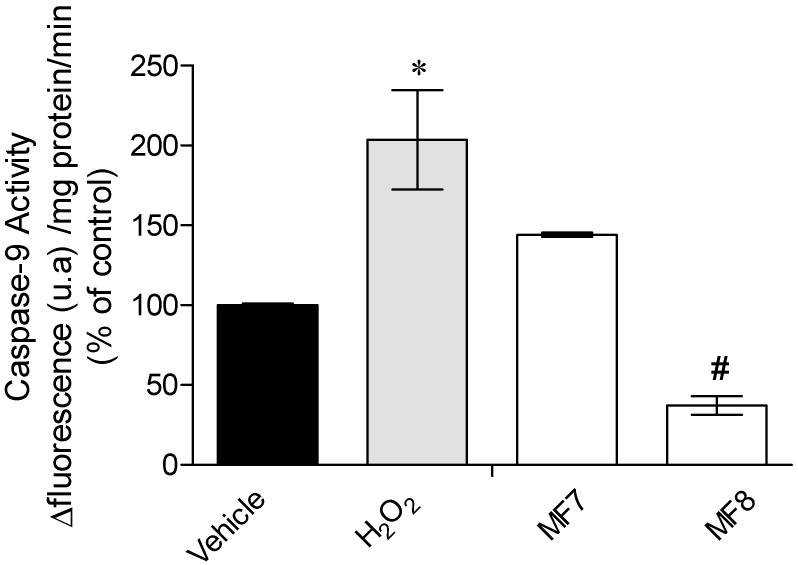
Effects of H_2_O_2_ (0.2 mM) in the presence or absence of *Sargassum muticum* fractions (1 mg/mL) on Caspase-9 activity of MCF-7 cells after 24 h of treatment. Results are presented in arbitrary units of fluorescence per mg protein per minute (% control). The values in each column represent the mean ± standard error of the mean (SEM) from three experiments. Symbols represent statistically significant differences (*p* < 0.05, ANOVA, Dunett’s test) when compared to: * vehicle and ^#^ H_2_O_2_.

It is possible to see that Caspase-9 activity is significantly increased in the presence of H_2_O_2_, and in the presence of *S. muticum* fraction MF8, Caspase-9 activity significantly decreased (Figure 4).

## 3. Discussion

### 3.1. Vacuum Liquid Chromatography for Bioactive Compounds Concentration

Seaweeds have been recognized as a healthy food ingredient, while there are also increasing evidence that its secondary metabolites can be used as new structures for drug development. *Sargassum muticum* has been widely used in traditional oriental medicine, but there is scarce science-based data available on its bioactive properties, specially data sustained on cellular models. On the other hand, since oxidative stress is associated with the development of numerous diseases, it is fairly pertinent to evaluate its potential to protect cells from oxidative stress conditions. *Sargassum muticum* was already evaluated for its potential antioxidant compounds production [8,27], but few studies have been conducted to concentrate and purify its bioactive molecules. Moreover, the lack of studies contributing to the understanding of the mechanisms of action is also worthy of note. Here, two extraction methods and subsequent subfractions were tested for their antioxidant potential and also their effective protection on an in vitro cellular model—the MCF-7 cells. The vacuum liquid chromatography did not effectively concentrate the antioxidant molecules, nevertheless, the total phenolic content increased in the MDF8 fraction comparing with the crude MD extract (MDE) (Table 1). This fraction also revealed a high scavenging capacity, yet losing potential in peroxyl radicals scavenging. Since the crude extract is a complex mixture of compounds, along the chromatography process, many compounds may be lost and others concentrated, which may justify the increase in the concentration of antioxidant molecules capable of detoxifying the DPPH radical. Concerning the methanolic extraction, the fractioning also promoted the reduction of the total phenolic content and the considerable loss of compounds with the capacity to scavenge peroxyl radicals, while the capacity of scavenging the DPPH radical was slightly increased. Not surprisingly, the last fractions of the VLC are the ones revealing the most interesting antioxidant activity, since many antioxidant compounds, including phlorotannins, have polar characteristics and therefore could be retained longer in the column [30]. 

### 3.2. Protective Effects of Sargassum muticum Enriched Fractions on an Oxidative Stress Condition Induced by H_2_O_2_

Since a previous study identified cytotoxic characteristics on *S. muticum* [27], it was important to understand the toxic effects on the human cellular model used in this work, the MCF-7 cells, since along the purification process it could be possible to separate non-toxic substances, conferring importance to the purification process. As a result, fractions MF2, MF3, MF5, MDF5, MDF6, and MDF8 presented toxicity and thus their cytoprotection was not tested. All other fractions were tested for their potential for protecting cells on an oxidative stress condition induced by H_2_O_2_.

H_2_O_2_ is one of the most widely used agents to induce oxidative stress on in vitro models [31]. This molecule is involved in many cellular mechanisms and presents high membrane permeability, allowing it to enter cells readily, inducing toxicity. Moreover, its relative stability enables a reproducible oxidative stress condition in the cells [32,33].

Fractions MF7 and MF8 presented capacity to reduce the H_2_O_2_ induced effects (Figure 1), which resulted in the increase of cell’s viability.

The following step involved the quantification of the cells H_2_O_2_ production, where it is possible to verify that the production of H_2_O_2_ was strongly inhibited by the presence of these enriched fractions (Figure 2). H_2_O_2_ is one of the main intercessors of oxidative stress-induced cytotoxicity [34]. Thus, the results strongly suggest that the increase of cell’s viability is related to the capacity of MF7 and MF8 to block the damage effects of H_2_O_2_, as well as the ability to decrease its production. Accordingly, other studies assessing the protective effects of seaweed have already revealed that these marine organisms contain compounds with capacity to protect cells from oxidative stress through H_2_O_2_-mediated disruption [8,35,36,37]. O’Sullivan and co-workers [38] also evaluated the capacity of seaweed to protect cellular damage induced by H_2_O_2_ and verified that *Fucus serratus* and *Fucus vesiculosus* (Fucales) reduced H_2_O_2_-mediated DNA damage. Kang and collaborators [36] also showed that phlorotannins from the brown seaweed *Ecklonia cava* produced neuroprotective effects in murine hippocampal HT22 cells against H_2_O_2_-induced oxidative stress. Moreover, the effects exhibited by MF7 and MF8 fractions become more relevant since they did not demonstrated cytotoxicity when tested in 3T3 cells. 

### 3.3. Mechanisms of Action Insight: Mitochondrial Membrane Potential and Caspase-9 Activity Evaluation

Concerning the effects of H_2_O_2_ in the mitochondrial membrane potential (ΔΨm), it can be observed that this induced a noticeable membrane depolarization (Figure 3). The mitochondrial membrane potential is essential to maintain the physiological function of the respiratory chain, and therefore changes on ΔΨm can promote cell death since it is involved in early apoptotic signaling pathways [28,29]. In the presence of MF7 fraction, the depolarization induced by H_2_O_2_ was maintained, but the MF8 fraction seemed to increase the H_2_O_2_ effects by increasing membrane polarization. These results suggest that the protective effects of these fractions are not directly associated to the mitochondrial membrane’s potential. Other possibility for the observed results was the involvement of the compounds in apoptotic mechanisms, and therefore, to deepen the protective effects of these fractions, the Caspase-9 activity was also assessed. Caspase-9 is one of the downstream regulators of apoptosis being associated to oxidative stress pathways [39]. Although the increase of ΔΨm is associated with the activation of caspases cascade, MF8 induced a marked reduction of more than 80% of Caspase-9 activity (Figure 4). Similarly, Chia and co-workers [40] evaluated the ability of seaweed to reduce cytotoxicity on MCF-7 cells and verified that *Turbinaria ornate* (also belonging to the Fucales order) also reduced Caspase-9 activity. Leeand co-workers [41] also showed that seaweed phlorotannins promoted cellular protection of HepG-2 cells by reducing Caspase-3 activity.

Together, the results suggest that *Sargassum muticum* contains compounds with the capacity to decrease cell’s oxidative stress levels by blocking the production of H_2_O_2_ and acting as downstream blockers of apoptosis. These characteristics now depicted, reveal *Sargassum muticum* potential as an excellent source of functional ingredients against oxidative stress conditions including those related with neurodegenerative diseases.

## 4. Material and Methods

### 4.1. Chemicals and Reagents

Methanol (MeOH) and dichloromethane (DCM) of analytical grade were purchased from Fisher Scientific (Loughborough, Leicestershire, UK). All the other chemicals and reagents, molecular and analytical grade, were purchased from Sigma (Sigma-Aldrich GmbH, Steinheim, Germany).

### 4.2. Collection, Preparation and Extraction of Sargassum muticum

*Sargassum muticum* specimens were collected at Praia Norte beach, Viana do Castelo, Portugal (41°41′44.2′′ N 8°51′08.1′′ W), washed with seawater to remove epibionts, sand and debris, frozen at −80 °C and then freeze-dried. Two independent extractions were carried out, one with 100% MeOH (M) and another with 1:1 MeOH:DCM (MD) as follows: freeze-dried samples were extracted overnight with constant stirring in a 1:40 biomass:solvent ratio. The crude extract was then filtered and evaporated in a rotary evaporator at 40 °C and the biomass stored at −20 °C until further use.

### 4.3. Fractionation of Sargassum muticum by Vacuum Liquid Chromatography (VLC)

To concentrate the bioactive compounds of each crude extract, a normal phase vacuum liquid chromatography (VLC) on silica gel 60 (0.06–0.2 mm) was performed. The solvent system (400 mL), of the methanolic crude extract, consisted in cyclohexane (F1) with increasing amounts (25%) of ethyl acetate, resulting in 5 fractions (MF1-MF5). The last 3 fractions were prepared with DCM), DCM: MeOH (1:1), and MeOH (MF6, MF7, and MF8, respectively).

For the 50:50 crude extract, the same column was used and the solvent system was the same for the first 6 fractions (MDF1-MDF6), however fraction MDF7 was eluted with 100% MeOH and fraction MDF8 was eluted with 50:50 MeOH:DCM.

Finally, all the solvents were evaporated and the dried fractions were stored at −20 °C until further use. The first elution (MF1) of the methanolic extraction did not resulted in enough biomass for the bioassays, and therefore the tests were assessed on MF2–MF8.

### 4.4. Analysis of Total Phenolic Content (TPC)

Total phenolic content of *S. muticum* fractions was determined using Folin–Ciocalteu method [42] adapted for microplate. Briefly, 2 µL of extract was added to 158 µL of distilled water and 10 µL of Folin–Ciocalteu reagent, vortexed and 30 µL of 20% Na_2_CO_3_ (*w*/*v*) was added. After one hour of reaction in the dark, the absorbance was measured at 755 nm (Synergy H1 Multi-Mode Microplate Reader, BioTek^®^ Instruments, Winooski, VT, USA) against a blank solution. The TPC is expressed as milligrams of phloroglucinol equivalents per gram of dry extract (mg PE g extract^−1^).

### 4.5. Evaluation of Antioxidant Activity

#### 4.5.1. DPPH (1,1-Diphenyl-2-picryl-hydrazyl) Radical Scavenging Activity

The DPPH radical scavenging activity was performed according to Brand-Williams and co-workers [43] adapted for microplate. The reaction occurred in the dark with 2 µL of each test sample (1000 μg/mL) and 198 µL of the DPPH solution (0.1 mM in ethanol). After 30 min incubation, the absorbance was measured at 517 nm. Butylated hydroxytoluene (BHT) was used as a standard. The scavenge potential was calculated in percentage of control. For the extracts that scavenged the DPPH radical in more than 50%, IC_50_ values (μg/mL) were determined.

#### 4.5.2. Oxygen Radical Absorbance Capacity (ORAC)

Oxygen radical absorbance capacity (ORAC-fluorescein assay) was evaluated as described by Dávalos and co-workers [44]. Briefly, the reaction was carried out in 75 mM phosphate buffer (pH 7.4), for a final reaction mixture of 200 µL. Sample (20 µL) and fluorescein (120 µL; 70 nM, final concentration) were placed in the wells of 96-well microplates and preincubated for 15 min at 37 °C. AAPH solution (60 µL; 12 mM, final concentration) was added and the fluorescence (λ_excitation_: 458 nm, λ_emission_: 520 nm) recorded every minute for 240 min. The microplate was automatically shaken prior to each reading. A blank, using phosphate buffer instead of the fluorescein, and eight calibration solutions using Trolox (1–8 µM, final concentration) were also carried out in each assay. All the reaction mixtures were prepared in duplicate, and at least three independent assays were performed for each sample. Antioxidant curves (fluorescence versus time) were first normalized to the curve of the blank, corresponding to the same assay by multiplying original data by the factor fluorescence_blank,t=0_/fluorescence_sample,t=0_. From the normalized curves, the area under the fluorescence decay curve (AUC) was calculated as:

$$\mathrm{AUC}\;=1\;+\;\overset{i=80}{\underset{i=1}{\sum }}\frac{f_{i}}{f_{0}}$$

where *f*_0_ is the initial fluorescence reading at 0 min and *f_i_* is the fluorescence reading at time *i*. Final results were expressed in µmol of Trolox equivalents/g of dry extract (µmol TE/g).

### 4.6. In Vitro Assay of Oxidative Stress Prevention

#### 4.6.1. Cell Maintenance Culture Conditions

Human breast adenocarcinoma model (MCF-7 cells—ACC 115) and murine fibroblasts (3T3 cells—ACC 173) were acquired from the German collection of microorganisms and cell cultures (DSMZ). All cells were maintained according DSMZ procedure.

#### 4.6.2. *Sargassum muticum* Fractions Cytotoxicity Evaluation

The cytotoxicity of seaweed fractions in both cell lines (MCF-7 and 3T3 cells) was determined after cells reached the total confluence in 96-well plates. Samples (1 mg/mL) were dissolved in RPMI 1640 medium without FBS, sterile filtered (0.2 µm) and incubated with cells during 24 h. Cell viability was then evaluated by the MTT method [45].

#### 4.6.3. Evaluation of the Protective Effect of *Sargassum muticum* Fractions on an Oxidative Stress Condition Induced by H_2_O_2_ on MCF-7 Cells

*Sargassum muticum* fractions without cytotoxicity were tested for their potential to prevent an oxidative stress condition induced on MCF-7 cells by the addition of H_2_O_2_ (0.2 mM). The concentration of the extracts and H_2_O_2_, and the assay conditions were defined in preliminary tests, and established as follows: after cells reached total confluence in 96-well plates, seaweed fractions (1 mg/mL) were added together with H_2_O_2_ (0.2 mM), during 24 h. The results were expressed in percentage of control.

#### 4.6.4. Real-Time Quantification of H_2_O_2_ Production

Quantification of H_2_O_2_ was assessed with the “Amplex^TM^ Red hydrogen peroxide Assay” Kit (Life Technologies, Carlsbad, CA, USA). The Amplex Red is a fluorophore that evidences a low basal fluorescence, which reacts with H_2_O_2_ in a 1:1 ratio. This reaction is initiated with horseradish peroxidase and successive reactions occur leading to the appearance of resofurin, a highly fluorescent product [46]. H_2_O_2_ production was quantified on MCF-7 cells after 24 h of treatment with H_2_O_2_ (0.2 mM) and seaweed fractions (1 mg/mL). The fluorescence was measured at excitation/emission 590/530 nm along 60 min, immediately after the addition of the fluorophore. The levels of H_2_O_2_ were calculated through the slope of the linear phase of fluorescence curve and the results expressed as percentage of control.

#### 4.6.5. Mitochondrial Membrane Potential (ΔΨm)

Mitochondrial membrane potential was determined using the fluorescent probe, JC-1 (Molecular Probes, Eugene, OR, USA). MCF-7 cells were treated with H_2_O_2_ (0.2 mM) in the absence or presence of seaweed fractions (1 mg/mL) during 24 h. Cells were then washed with Hank’s buffer and incubated with JC-1 (3 µM) during 15 min at 37 °C. The formation of JC-1 aggregates (excitation/emission 490/590 nm) and the monomeric form of JC-1 (excitation/emission 490/530 nm) was accompanied simultaneously during 30 min. Results were expressed as the ratio of the monomers/aggregates of JC-1 as percentage of control.

#### 4.6.6. Caspase-9 Activity

Caspase-9 activity was assessed using the “Caspase-9 Assay kit” K118 (Biovision, Milpitas, CA, USA). Cells were cultured in six-well plates and treated with H_2_O_2_ (0.2 mM) and *S. muticum* fractions (1 mg/mL—24 h), washed twice with Hank’s buffer, collected by centrifugation (2300 *g*, 10 min, 4 °C), resuspended in 50 µL of lysis buffer, incubated 20 min on ice and centrifuged (15,870 *g*, 20 min, 4 °C). After, 50 µL of supernatant was transferred to a 96-well plate, and added to 50 µL of reaction buffer containing dithiothreitol (10 mM) and 5 µL of substrate. This reaction was followed at excitation/emission: 400/505 nm along 90 min. Caspase-9 activity was calculated through the slope of the fluorescence resulting from 7-amino-4-(trifluoromethyl) coumarin accumulation and expressed as % of control (Δ fluorescence (u.a)/mg of protein/min).

### 4.7. Statistical Analysis

The IC_50_ concentration was calculated from nonlinear regression analysis using GraphPad Prism software with the equation: Y = 100/(1 + 10^(X−LogIC^_50_^)^). All data were checked for normality and homoscedasticity using the Shapiro–Wilk and Levene’s test, respectively. Comparisons concerning variables, which did not meet variance or distributional assumptions, were carried out with the Kruskal–Wallis nonparametric tests [47]. One-way analysis of variance (ANOVA) was carried out when evaluating the effects of seaweed fractions (1 mg/mL) in an oxidative stress condition promoted by H_2_O_2_ addition (0.2 mM) on MCF-7 cells after 24 h of incubation. The statistical comparisons among the groups were performed with the Newman–Keuls multiple comparison test [47]. For all statistical tests, the significance level was set at *p*-value < 0.05 and results were expressed as mean ± standard error of the mean. All calculations were performed on GraphPad InStat v. 3.5 (GraphPad Software, La Jolla, CA, USA).

## Figures and Tables

**Table 1:** Total phenolic content and antioxidant activity of *Sargassum muticum* crude extract and vacuum liquid chromatography (VLC) fractions.

Fraction	TPC ^a^	DPPH ^b^	ORAC ^c^
Crude MDE	75.754 ± 0.026	28.42 (26.88–30.04)	12,634.197 ± 312.511
MDF1	25.786 ± 0.015	>1000	1.456 ± 0.531
MDF2	55.867 ± 0.025	>1000	16.136 ± 0.330
MDF3	38.854 ± 0.017	>1000	29.088 ± 5.833
MDF4	34.309 ± 0.006	>1000	11.353 ± 3.432
MDF5	60.056 ± 0.024	57.33 (51.63–63.66)	280.989 ± 13.153
MDF6	36.779 ± 0.008	81.13 (67.95–96.87)	136.825 ± 14.404
MDF7	21.668 ± 0.008	98.84 (84.62–115.5)	770 ± 26.332
MDF8	84.080 ± 0.026	32.29 (28.95–36.02)	3040.143 ± 266.235
Crude ME	85.256 ± 1.158	53.1 (46.7–67.82)	2672.80 ± 54.22
MF2	20.447 ± 0.964	106.4 (92.9–121.9)	14.662 ± 0.514
MF3	16.860 ± 0.565	291.0 (261.9–323.3)	17.458 ± 0.152
MF4	25.619 ± 1.053	>1000	42.489 ± 0.895
MF5	26.653 ± 0.831	>1000	77.877 ± 22.648
MF6	11.663 ± 0.413	>1000	42.892 ± 0.190
MF7	36.723 ± 1.314	>1000	44.404 ± 15.050
MF8	61.668 ± 1.496	36.4 (32.4–41.0)	51.975 ± 4.280
BHT	-	50.3 (36.0–54.3)	330.69 ± 37.52

**^a^** mg Phloroglucinol equivalents/g extract (PE/g); ^b^ DPPH radical scavenging activity (IC_50_ µg/mL); ^c^ µmol Trolox equivalents/g extract (TE/g); MD—methanol:dichloromethane; M—Methanol; E—extract.

## Supplementary Materials

The following are available online at www.mdpi.com/1660-3397/15/11/353/s1, Figure S1: Evaluation of Sargassum muticum fractions cytotoxicity on MCF-7 cells (1 mg/mL; 24 h). Results were obtained by the MTT method. Values in each column are the mean of 3 independent experiments ± standard error of the mean (SEM). Symbols (*) represent statistically significant differences (*p* < 0.05, ANOVA, Dunett’s test) when compared to vehicle.
