# Supplementary material for: An Insight into Sargassum muticum Cytoprotective Mechanisms against Oxidative Stress on a Human Cell In Vitro Model
Source: Mar Drugs. 2017 Nov 10;15(11):353. doi: 10.3390/md15110353 (PMC5706042; doi:10.3390/md15110353)
Supplement: Supplementary file 1 [file marinedrugs-15-00353-s001.pdf]

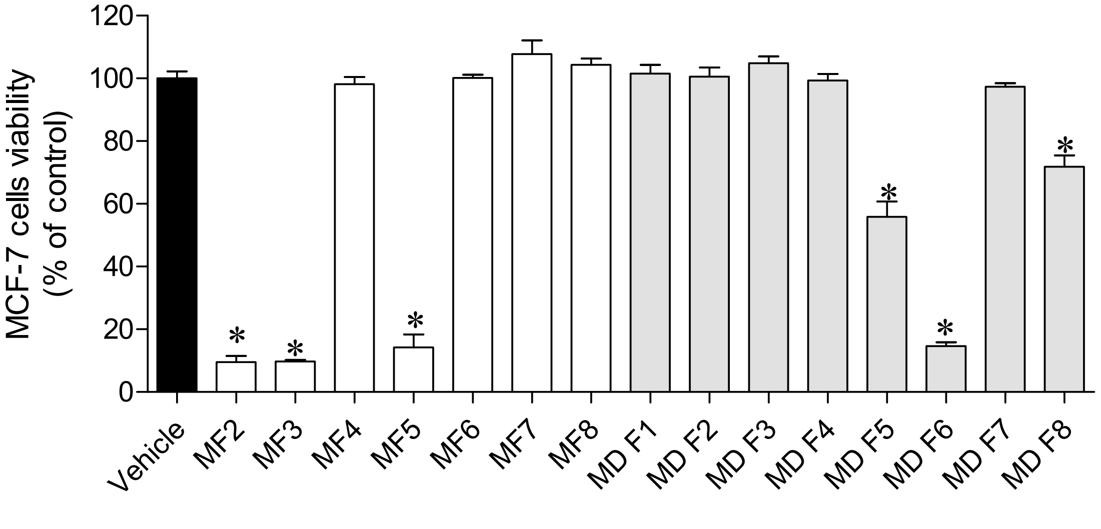

Figure 1 – Evaluation of *Sargassum muticum* fractions cytotoxicity on MCF-7 cells (1 mg/mL; 24 h). Results were obtained by the MTT method. Values in each column are the mean of 3 independent experiments ± standard error of the mean (SEM). Symbols (*) represent statistically significant differences ($p < 0.05$, ANOVA, Dunett's test) when compared to vehicle.
